# A method dealing with a large number of correlated traits in a linkage genome scan

**DOI:** 10.1186/1753-6561-1-s1-s84

**Published:** 2007-12-18

**Authors:** Tao Feng, Shuanglin Zhang, Qiuying Sha

**Affiliations:** 1 Department of Mathematical Sciences, Michigan Technological University, 2011B Woodmar Drive, Houghton, Michigan 49931, USA; 2 Department of Mathematics, Heilongjiang University, Harbin 150080, China

## Abstract

We propose a method to perform linkage genome scans for many correlated traits in the Genetic Analysis Workshop 15 (GAW15) data. The proposed method has two steps: first, we use a clustering method to find the tight clusters of the traits and use the first principal component (PC) of the traits in each cluster to represent the cluster; second, we perform a linkage scan for each cluster by using the representative trait of the cluster. The results of applying the method to the GAW15 Problem 1 data indicate that most of the traits in the same cluster have the same regulators, and the representative trait measure, the first PC, can explain a large part of the total variation of all the traits in each cluster. Furthermore, considering one cluster of traits at a time may yield more linkage signals than considering traits individually.

## Background

From yeast to humans, the expression level of many genes shows familial aggregation and a simple segregation pattern in [1-3], suggesting an inherited contribution. Morley et al. [3] used microarray to measure the baseline expression level of genes in immortalized B cell from members of 14 Centre d'Etude du Polymorphism Humain (CEPH) pedigrees. They mapped the quantitative trait loci (QTL) of many quantitative traits (gene expression phenotypes) to chromosomal locations using genome scans. The data set of Morley et al. was made available to us by Genetic Analysis Workshop 15 (GAW15). This data set provides genome scan data for 3554 quantitative traits, or expression phenotypes of 3554 genes.

Morley et al. carried out a linkage genome scan for each of the 3554 traits individually and found that 142 and 984 of the 3554 traits have at least one significant linkage signal when the point-wise *p*-value thresholds 4.3 × 10^-7 ^and 3.7 × 10^-5 ^were used, respectively. Considering the correlated nature of the 3554 traits, we proposed a method to find clusters of genes whose expressions appear to be highly correlated and to localize the genetic determinants of each of the clusters. The proposed method has two steps: 1) use a clustering method to find tight clusters of the traits, and in each cluster, use the first principal component (PC) of the traits to represent this cluster; and 2) carry out a linkage scan using the first PC trait for each cluster and consider the linkage evidence of the first PC trait as the linkage evidence of all the traits in the cluster. Upon applying the method to the GAW15 Problem 1, we found that: 1) the traits in the same cluster have linkage signals at the same chromosomal regions; 2) the first PC can explain more than 53% of the total variation of all the traits in each cluster, which indicates that it is reasonable to represent a tight cluster of traits by using the first PC; and 3) this clustering of highly correlated traits may show more linkage signals than considering the traits individually.

## Methods

### Tight clustering

Tight clustering, first proposed by Tseng and Wong [4], is a method that produces tight and stable clusters without forcing all points into clusters. Tseng and Wong used a resample-based algorithm. Briefly, in each resample, the *K*-mean clustering algorithm is used to group the sampled genes into clusters. A similarity measure between two genes is defined as the number of resamples in which the two genes are in the same cluster. Then, this similarity measure is used to define a tight cluster. Here, to find a cluster of highly correlated genes, we propose a new tight clustering algorithm, which is computationally simpler than Tseng and Wong's algorithm.

Suppose there are *n *individuals and each individual has *m *traits (expressions of *m *genes). Let *x*_*ij *_denote the trait value of the *j*^th ^trait of the *i*^th ^individual, and *x*_*j *_= (*x*_1*j*_,..., *x*_*nj*_) denote the values of the *j*^th ^trait of all individuals. A set of traits is said to be a tight cluster if the average correlation coefficient between traits in this set is larger than a given threshold *α*, and the number of traits in this set is between *m*_1 _and *m*_2_. Briefly, for given parameters *α*_0_, *m*_1 _and *m*_2_, our "tight clustering" algorithm involves the following steps: 1) for a given *α *(≥ *α*_0_), find the largest tight cluster. If a tight cluster for the given *α *cannot be found, reduce the value of *α*. If a tight cluster still cannot be found when *α *= *α*_0_, stop the algorithm. 2) Remove the traits in the tight cluster from the data set and repeat Step 1.

For a given data set and the values of the parameters *α*_0_, *m*_1 _and *m*_2_, it is a computational challenge to search the whole state space to find the largest tight cluster. We propose to use a backwards elimination approach to find an approximate solution. Let *ρ*_*j *_denote the average correlation coefficient between the *j*^th ^trait and all the other traits. The backward elimination has the following three steps: 1) rank the traits by *ρ*_*j *_from the largest to the smallest and delete a proportion (5% in our implementation) of traits with the smallest values of *ρ*_*j*_. 2) Recalculate *ρ*_*j *_in the new data set (5% of traits were deleted) and repeat Step 1. 3) Repeat Steps 1 and 2 until the average correlation coefficient is larger than *α *and the number of traits is between *m*_1 _and *m*_2_.

In our implementation, we used *m*_1 _= 20, *m*_2 _= 200, and *α*_0 _= 0.5 (see Discussion section for the effect of different parameter values). We first chose *α *= 0.8, and then reduced *α *to 0.7, 0.6, and 0.5. We then found the first PC of each cluster, which, because traits within are highly correlated, should explain a large part of the total variation.

### Linkage genome scan

We used the program *Merlin-regress *[5] to perform the linkage genome scan for each quantitative trait. *Merlin-regress *implements a method proposed by Sham et al. [6], which is based on a regression of estimated identity-by-descent (IBD) sharing between relative pairs on the squared sums and squared differences of trait values of the relative pairs. This program allows quick computation and has similar power of variance-component approaches.

The program *Merlin-regress *produces a *p*-value for each trait-marker pair. A linkage signal is declared if the *p*-value is less than a cut-off value. We propose to obtain a cut-off value of individual *p*-values by controlling the false-discovery rate (FDR). Suppose we have *M *trait-marker pairs and denote the ordered *p*-values by *p*_(1)_,..., *p*_(*M*)_. To control the FDR at 5%, the cut-off value of the individual *p*-value will be as described by Benjamini and Yekutieli [7]:

$$\beta_{0}=\underset{i}{\max}\left\{p_{\left(i\right)}:p_{\left(i\right)}\leq 0.05i/(MC_{M})\right\},\text{ where }C_{M}=\sum_{i=1}^{M}1/M.$$

## Results

We applied the proposed method to the data set given by the GAW15 Problem 1. The data set contained members of 14 CEPH Utah families. Each individual had 3554 traits and genotypes at 2882 SNPs across the genome. We deleted some SNPs with genotypes that showed Mendelian inconsistency. There were 2761 SNPs left for further analysis.

**Figure 1:**
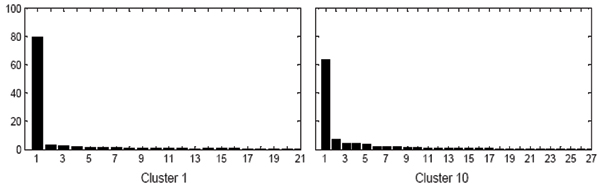
Eigenvalues (in percentage of the sum of all eigenvalues) of cluster 1 and cluster 10.

Upon applying the tight clustering algorithm to 3554 traits, we found 18 clusters with an average correlation coefficient larger than 0.5. There were 734 traits, ~20% of the total traits, in the 18 clusters. The details of the 18 clusters are given in Table 1. We obtained one cluster with average correlation of ~0.8, four clusters with average correlation of ~0.7, five clusters with average correlation of ~0.6, and eight clusters with average correlation of ~0.5. Then, we applied PC analysis to each of the clusters. The eigenvalues of clusters 1 and 10 are shown in Figure 1. This figure shows that the first eigenvalue is much larger than the other eigenvalues. The eigenvalues of other clusters show a similar pattern. The ratio of the first eigenvalue to the sum of all eigenvalues is approximately equal to the average correlation coefficient of the cluster and is more than 50% in all clusters. That is, the first PC explains more than 50% of the total variation for all clusters. Thus, the first PC is a reasonable measure to use to represent all the traits in each cluster.

**Table 1:** The 18 clusters found by the tight clustering method

**Cluster ID**	**# of traits**	**Average correlation**
1	21	0.8
2	43	0.7
3	32	0.7
4	27	0.7
5	28	0.7
6	35	0.6
7	35	0.6
8	47	0.6
9	33	0.6
10	27	0.6
11	64	0.5
12	42	0.5
13	84	0.5
14	59	0.5
15	44	0.5
16	41	0.5
17	37	0.5
18	44	0.5

We carried out a linkage scan for each of the 18 first PC traits by using the program *Merlin-regress*. For comparison, we also performed a linkage scan for each of the 743 traits in the 18 clusters. For a linkage genome scan, the program *Merlin-regress *gave *p*-values of the 2761 SNPs. We used the Eq. (1) to find the cut-off value *β*_0 _of the individual *p*-values to control the FDR at 5%. For the 18 PC traits, we got *M *= 18 × 2761 trait-marker pairs and the cut-off value *β*_0 _= 1.67 × 10^-5^. For the 743 individual traits in the 18 clusters, there were *M *= 743 × 2761 trait-marker pairs, and the cut-off value was *β*_0 _= 5.34 × 10^-8^.

**Figure 2:**
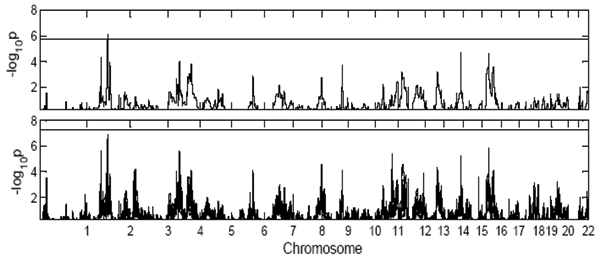
The figure in the top panel shows the *p*-value curve for the first PC trait of the cluster. The figure in the bottom panel shows *p*-value curves for the individual traits of the cluster (total 28 traits in cluster 5).

Following Morley et al. [3], the regions with linkage signals (*p*-value less than the cut-off value) are considered to be regulators. The number of regulators of each of the 18 PC traits and the number of traits with at least one regulator in each cluster are summarized in Table 2. Table 2 shows that 6 out of 18 (33%) PC traits have at least one regulator, while 16 out of 743 (2.2%) individual traits have at least one regulator. We conclude that considering one cluster at a time may show more linkage signals than considering traits individually. Figure 2 gives *p*-value curves of PC traits and individual traits in cluster 5. The figure indicates that the linkage peaks of all individual traits and the PC trait of the cluster appear to be very close to each other. It indicates that the traits in one cluster have similar regulators, and we can find these regulators by using the PC trait. Furthermore, we can see from Table 2 that in 16 out of 18 clusters, if the PC trait has regulators, there will be some individual traits with regulators, and vice versa. This indicates that the PC trait is a reasonable measure to represent all the traits in the corresponding cluster.

**Table 2:** Details of the 18 clusters.

Cluster ID	No. regulators for the PC trait	No. traits	No. RE traits^a^
1	1	21	0
2	0	43	0
3	2	32	6
4	0	27	0
5	1	28	0
6	0	35	0
7	0	35	0
8	2	47	5
9	0	33	0
10	2	27	1
11	0	64	0
12	0	42	0
13	0	84	2
14	1	59	2
15	0	44	0
16	0	41	0
17	0	47	0
18	0	44	0

^a^RE trait is the trait with at least one regulator.

## Discussion

In order to analyze a large number of correlated traits given in the GAW15 Problem 1, we have developed a method that first clusters the traits into tight clusters and then finds a representative measure for each cluster. We then performed a linkage scan for the representative trait. The results of analyzing the GAW15 Problem 1 data set indicate that the traits in the same cluster have the same regulators, and that the first PC in one cluster is a good representative measure of this cluster. The results also show that performing a genome scan for one cluster at a time may show more linkage signals than that for genome scans of individual traits.

One remaining problem is how to choose the values of parameters *α*_0_, *m*_1_, and *m*_2 _in the tight clustering algorithm. We have applied the algorithm to the GAW15 data with different parameter values. Our results indicate that when *m*_2 _varies from 100 to 1000, there is almost no effect on the cluster assignment and linkage analyses. When *m*_1 _is small, say 2, there will be more small clusters and more linkage signals. Because two biologically unrelated genes may have highly correlated traits by chance, by using a small *m*_1_, the tight clustering algorithm may cluster biologically unrelated genes into one cluster. We suggest choosing *m*_1 _between 10 and 20, and *m*_2 _between 100 and 400. Our independent simulation studies indicate that if *α*_0 _is too small, the FDR cannot be controlled, and the first PC trait will not be a good representative of the cluster, because the first PC can only explain a small part of the total variation. Thus we suggest using *α*_0 _≥ 0.5.

## Competing interests

The author(s) declare that they have no competing interests.
